# Modeling the influence of Twitter in reducing and increasing the spread of influenza epidemics

**DOI:** 10.1186/s40064-016-1689-4

**Published:** 2016-01-27

**Authors:** Hai-Feng Huo, Xiang-Ming Zhang

**Affiliations:** Department of Applied Mathematics, Lanzhou University of Technology, Lanzhou, 730050 Gansu People’s Republic of China

**Keywords:** Positive effect and negative effect, Twitter, Basic reproductive number, Equilibria, Global stability, Hopf bifurcation, 92D25

## Abstract

A more realistic mathematical influenza model including dynamics of Twitter, which may reduce and increase the spread of influenza, is introduced. The basic reproductive number is derived and the stability of the steady states is proved. The existence of Hopf bifurcation are also demonstrated by analyzing the associated characteristic equation. Furthermore, numerical simulations and sensitivity analysis of relevant parameters are also carried out. Our results show that the impact posed by the negative information of Twitter is not significant than the impact posed by the positive information of Twitter on influenza while the impact posed by the negative information of Twitter on the influenza virus is still extraordinary.

## Background

Twitter is an online social networking service that enables users to send and read short 140-character messages called “tweets”. The service has gained worldwide popularity rapidly since it launched in 2006 (Dijck 2011; Elsweiler and Harvey 2015; Dugue and Perez 2014). Roughly speaking, the site is largely used to daily chatter, conversation, and debate about information, or to allow ones to understand the world by Daily News (Java et al. 2007). Twitter’s users who are increasingly diverse in age, ethnicity and gender may see the messages of other followers and go along with these users (Sanderson 2014). Reciprocally, nicknames that follow a user have the ability to see the messages posted by other users (Dugue and Perez 2014). By doing this, it is fairly easy to make up a following of 20–30 people quickly (Launer 2013). At the same time, these companions who make full use of Twitter are strongly influenced by minority status, party leadership efforts, chamber, and member age (Lassen and Brown 2011). Twitter becomes so popular nowadays not only because of it can provide positive information (Tiernan 2014; Fu and Shen 2014; Roshanaei and Mishra 2015) but also the negative information (Jin et al. 2014; Dugue and Perez 2014; Alowibdi et al. 2015; Roshanaei and Mishra 2015) it still can be provided. As of June 2013, Twitter had 218 million monthly active users who collectively expressed around 500 million tweets a day (Elsweiler and Harvey 2015). Undoubtedly, Twitter has become a more and more powerful tool for spreading and mining messages in our daily life (Fu and Shen 2014).

Social networking sites play a vital role in medicine and other walks of modern life. Public Health Organizations (WHO), Centers for Disease Control and Prevention (CDC), the Food and Drug Administration (FDA), and the American Red Cross always increasingly advocate public to take advantage of social media programs included Twitter, Facebook, and similar internet sites to disseminate important health information. For example, the CDC made full use of Twitter to post messages for preventing flu to help slow the spread of H1N1 influenza in 2009, growing from 2500 followers to 370,000 followers during the 2009 outbreak (Currie 2009). It is observed that information that users of Twitter shared took advantages or disadvantages for spreading of infectious diseases by reminding them to stay at home when they are sick, teaching users the effectiveness of regular hand-washing, and raising awareness about vaccines or misleading their do some irrational things.

Influenza has always a far-reaching influence on our lives, and many attempts have been made to investigate realistic mathematical models for researching the transmission dynamics of infectious diseases (Cui et al. 2008b; Xiao et al. 2013; Sahua and Dhara 2015; Wang et al. 2015; Kaur et al. 2014; Misra et al. 2011; Liu and Cui 2008; Cui et al. 2008a; Pawelek et al. 2014; Liu et al. 2007). Cui et al. (2008) proposed a SIS-type model to explore the influence of media coverage on the dissemination of emerging or reemerging infectious disease, and used a standard incidence $\frac{{\beta SI}}{{S + I}}$ between susceptible individuals and infected individuals. Their results indicated that media coverage was critical for educating people in understanding the possibility of being infected by the disease. Xiao et al. (2013) developed a model with media coverage by including a piecewise smooth incidence rate to show that the reduction factor due to media coverage relies on both the number of cases and the rate of changes in case number. They demonstrated that the media impact resulted in a lower size of outbreak and delayed the epidemic peak. Liu and Cui (2008) considered a epidemic model with non-linear contact rate, $\beta (I) = {\beta _1} - {\beta _2}\frac{I}{{m + I}}$, where $\beta _1$ is the contact rate before media alert, and $\beta (I)$ is the contact rate after media alert, and studied the basic reproductive number, the existence and stability of two equilibria. They showed that media and education played a crucial role in mounting infection awareness among the residents. An exponential incidence $\beta (I) = \mu {e^{ - mI}}$ was applied to develop a three dimensional compartmental model Cui et al. (2008a). They analyzed dynamical behavior of the model; permanent oscillations are generated by a Hopf bifurcation. Pawelek et al. (2014) developed a simple mathematical model including the dynamics of “tweets”, and studied dynamics of the model. They showed that Twitter may serve as a good indicator of seasonal influenza epidemics. Liu et al. (2007) assumed that the total number of susceptible remains relatively unchanged as a result of the outbreak duration is extremely short, and incorporated a simple nonlinear incidence function ${\beta _0} = \beta {e^{ - {\alpha _1}E - {\alpha _2}I - {\alpha _3}H}}$, where *H* denotes hospitalized individuals. They illustrated the multiple outbreaks or the sustained periodic oscillations of emerging infectious diseases owing to the psychological impact.

It is well known that everything has two sides in reality. Massive media coverage is no exception. Alowibdi et al. (2015) focused specifically on the detection of inconsistent information involving user gender and user location; they shown that lying contained misleading, inconsistent, or false and deceptive information in online social networks is quite widespread. Roshanaei and Mishra (2015) compared the patterns of tweeting, replying and following by analysis of social engagement and psychological process in the positive and negative networks; their findings not only predicted positive and negative users but also provided the best recommendation for negative users through online social media. Unfortunately, most of the aforementioned model (Cui et al. 2008b; Sahua and Dhara 2015; Wang et al. 2015; Kaur et al. 2014; Misra et al. 2011; Liu and Cui 2008; Cui et al. 2008a; Pawelek et al. 2014; Liu et al. 2007) ignored the negative role of the media coverage. It has been observed that communications that people received or send through Twitter mislead the public to do some irrational things as well as benefited some people (Tiernan 2014; Fu and Shen 2014; Jin et al. 2014; Dugue and Perez 2014). Inspired by the documents (Cui et al. 2008a; Liu and Cui 2008; Liu et al. 2007; Pawelek et al. 2014), we introduce a more realistic mathematical influenza model, which incorporates the effects of Twitter in reducing and increasing the spread of influenza epidemics.

The rest of the paper is organized as follows: In “Basic properties” section, a more realistic $SEI{T_1}{T_2}$ model is formulated, the basic reproductive number and stability of equilibria are also obtained. In “Analysis of the model” section, the Hopf bifurcation is studied. Numerical simulations are carried out in “Numerical simulation” section. Sensitivity analysis is conducted in “Sensitivity analysis” section. Some discussions and conclusions are given in the last section.

## Basic properties

### System description

**Fig. 1:**
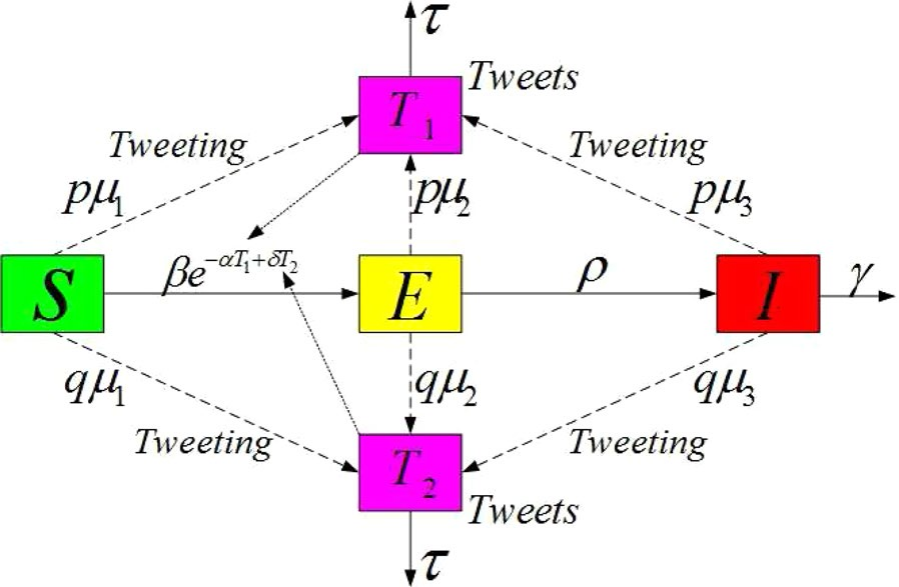
Transfer diagram for the dynamics of flu model

The total population is divided into three compartments: *S*(*t*), the number of susceptible individuals; *E*(*t*), the number of individuals exposed to the infected but not infectious; *I*(*t*), the infected who are infectious. All of them may tweet about influenza at the rates $\mu _1$, $\mu _2$, and $\mu _3$, respectively, during an epidemic season. $T_1(t)$ and $T_2(t)$ represent the number of tweets that all of them provide positive and negative information about influenza at time *t*, respectively. Our model is governed by the following system of five differential equations. A transfer diagram of our model is shown in Fig. 1 and the parameters description of our model are presented in Table 1.

**Table 1:** The parameters description of the flu model

Parameter	Description
$\beta$	Transmission coefficient from the susceptible compartment to the exposed compartment
$\alpha$	The coefficient that determines how effective the positive flu information can reduce the transmission rate
$\delta$	The coefficient that determines how effective the negative flu information can increase the transmission rate
$\rho$	Transmission coefficient from the exposed compartment to the infected compartment
$\gamma$	The permanently recover rate
$\mu _{i},i=1,2,3$	The rate that susceptible individuals, exposed individuals, and infectious individuals may tweet about influenza during an epidemic season respectively
*p*	The ratio that individuals may provide positive information about influenza during an epidemic season
*q*	The ratio that individuals may provide negative information about influenza during an epidemic season
$\tau$	The rate that tweets become outdated

The transfer diagram leads to the following system of ordinary differential equations:

$$\begin{aligned} \frac{{dS(t)}}{{dt}} &= - \beta SI{e^{ - \alpha {T_1} + \delta {T_2}}},\\ \frac{{dE(t)}}{{dt}}&= \beta SI{e^{ - \alpha {T_1} + \delta {T_2}}} - \rho E,\\ \frac{{dI(t)}}{{dt}}&= \rho E - \gamma I,\\ \frac{{d{T_1}(t)}}{{dt}}&= p{\mu _1}S + p{\mu _2}E + p{\mu _3}I - \tau {T_1},\\ \frac{{d{T_2}(t)}}{{dt}}&= q{\mu _1}S + q{\mu _2}E + q{\mu _3}I - \tau {T_2}, \end{aligned}$$

where all the parameters are positive constants and $\rho$ is the transmission coefficient from the exposed individuals to the infectious individuals, $\gamma$ is the recover rate that infectious individuals gain permanent immunity to that strain of influenza, *p* is the ratio that individuals may provide positive information about influenza during an epidemic season. *q* is the ratio that individuals may provide negative information about influenza during an epidemic season. For simplicity, we assume that the ratio of positive/negative information for all three groups is same, that is, *p* and *q*. $\mu _{i},i = 1,2,3$ is the rate that susceptible individuals, exposed individuals, and infectious individuals may tweet about influenza during an epidemic season, respectively. $\tau$ is the rate that tweets become outdated in consequence of tweets that appeared earlier are less visible and have less effect on the public, and $\beta$ is the disease transmission coefficient. The transmission coefficient $\beta$ is reduced by a factor ${e^{ - \alpha {T_1}}}$ owing to the behavior change of the public after reading positive tweets about influenza, where $\alpha$ determines how effective the disease positive twitter information can reduce the transmission coefficient, and is increased by a factor ${e^{ \delta {T_2}}}$ due to the behavior change of the public after reading negative tweets about influenza, where $\delta$ determines how effective the disease negative twitter information can increase the transmission coefficient. Since we only consider the disease outbreak during extremely short time, we neglect the natural death and birth rates and further assume that the number of susceptible people is relatively constant (Liu et al. 2007). Therefore, the above system can be reduced as follows:

1 $$\begin{aligned} \frac{{dE(t)}}{{dt}}&= \beta SI{e^{ - \alpha {T_1} + \delta {T_2}}} - \rho E,\nonumber \\ \frac{{dI(t)}}{{dt}}&= \rho E - \gamma I,\nonumber \\ \frac{{d{T_1}(t)}}{{dt}}&= p{\mu _1}S + p{\mu _2}E + p{\mu _3}I - \tau {T_1},\nonumber \\ \frac{{d{T_2}(t)}}{{dt}}&= q{\mu _1}S + q{\mu _2}E + q{\mu _3}I - \tau {T_2}. \end{aligned}$$

### The basic reproductive number

According to system (1), we can easily obtain the basic reproductive number $R_0$ by using the the next-generation method (Driessche and Watmough 2002). Here, we have the following matrix of new infection ${\mathcal {F}}(x)$, and the matrix of transfer ${\mathcal {V}}(x)$.

2 $${E_0} = (0,0,{T_1}^0,{T_1}^0) = \left( 0,0,\frac{{p{\mu _1}S}}{\tau },\frac{{q{\mu _1}S}}{\tau }\right).$$

Let $x=(E,\;I,\;T_1,\;T_2)^{T}$, then system (1) can be written as

$$\frac{dx}{dt}={\mathcal {F}}(x)-\mathcal {V}(x),$$

where

$$\begin{aligned} {\mathcal {F}}(x) &= \left( \begin{array}{c} {\beta SI{e^{ - \alpha {T_1} + \delta {T_2}}}}\\ 0\\ 0\\ 0\end{array} \right) ,\\ {\mathcal {V}}(x) &= \left( \begin{array}{c} {\rho E}\\ {\gamma I - \rho E}\\ {\tau {T_1} - p{\mu _1}S - p{\mu _2}E - p{\mu _3}I}\\ {\tau {T_2} - q{\mu _1}S - q{\mu _2}E - q{\mu _3}I} \end{array} \right) . \end{aligned}$$

The Jacobian matrices of ${\mathcal {F}}(x)$ and ${\mathcal {V}}(x)$ at the disease-free equilibrium $E_{0}$ are, respectively,

$$\begin{aligned} &D{\mathcal {F}}(E_{0}) = \left( \begin{array}{cccc} 0&{}{\beta S{e^{ - \alpha {T_1}^0 + \delta {T_2}^0}}}&{}0&{}0\\ 0 &{} 0 &{} 0 &{} 0\\ 0 &{} 0 &{} 0 &{} 0\\ 0 &{} 0 &{} 0 &{} 0\\ \end{array} \right) ,\qquad D{\mathcal {V}}(E_{0})=\left( \begin{array}{cccc} \rho &{}0&{}0&{}0\\ { - \rho }&{}\gamma &{}0&{}0\\ { - p{\mu _2}}&{}{ - p{\mu _3}}&{}\tau &{}0\\ { - q{\mu _2}}&{}{ - q{\mu _3}}&{}0&{}\tau \end{array} \right) ,\\ & D{\mathcal {V}}(E_{0})^{-1} = {} \left( \begin{array}{cccc} {\frac{1}{\rho }}&{}0&{}0&{}0\\ {\frac{1}{\gamma }}&{}{\frac{1}{\gamma }}&{}0&{}0\\ {\frac{{p{\mu _2}}}{{\tau \rho }} + \frac{{p{\mu _3}}}{{\tau \gamma }}}&{}{\frac{{p{\mu _3}}}{{\tau \gamma }}}&{}{\frac{1}{\tau }}&{}0\\ {\frac{{q{\mu _2}}}{{\tau \rho }} + \frac{{q{\mu _3}}}{{\tau \gamma }}}&{}{\frac{{q{\mu _3}}}{{\tau \gamma }}}&{}0&{}{\frac{1}{\tau }} \end{array} \right) . \end{aligned}$$

The basic reproductive number, denoted by $R_{0}$ is thus given by

3 $$R_{0}=\frac{{\beta S}}{\gamma }{e^{ - \alpha {T_1}^0 + \delta {T_2}^0}},$$

where

4 $${T_1}^0 = \frac{{p{\mu _1}S}}{\tau },\quad {T_2}^0 = \frac{{q{\mu _1}S}}{\tau }.$$

### The existence of equilibria

#### **Theorem 1**

*For the system (*1*), there exist the following two equilibria:*

1. *System* (1) *always exists the disease-free equilibrium*${E_0} = (0,0,{T_1}^0,{T_2}^0)$, *where*${T_1}^0$*and*${T_2}^0$*are given by* (4).
2. If $R_{0}>1$*and*$\alpha p - \delta q > 0$, *there exists the endemic equilibrium*${E_*} = ({E^*},{I^*},{T_1}^*,{T_2}^*)$. *Where*${E_*} = ({E^*},{I^*},{T_1}^*,{T_2}^*)$*satisfies the following equalities*:
  5 $$\begin{aligned} {E^*} &= \frac{{\gamma \rho \ln ({R_0})}}{{(\alpha p - \delta q)(\gamma {\mu _2} + \rho {\mu _3})}},\nonumber \\ {I^*} &= \frac{{\tau \rho \ln ({R_0})}}{{(\alpha p - \delta q)(\gamma {\mu _2} + \rho {\mu _3})}},\nonumber \\ {T_1}^* &= \frac{{p\ln ({R_0})}}{{(\alpha p - \delta q)}} + {T_1}^0,\nonumber \\ {T_2}^* &= \frac{{q\ln ({R_0})}}{{(\alpha p - \delta q)}} + {T_2}^0. \end{aligned}$$

#### *Proof*

1. It is easy to know that system (1) always exists the disease-free equilibrium $E_{0}=(0,0,\frac{{p{\mu _1}S}}{\tau },\frac{{q{\mu _1}S}}{\tau })$.
2. By letting the right-hand sides of (1) equal zero, namely,
  6 $$\begin{aligned}&\beta SI{e^{ - \alpha {T_1} + \delta {T_2}}} - \rho E = 0,\nonumber \\&\rho E - \gamma I = 0,\nonumber \\&p{\mu _1}S + p{\mu _2}E + p{\mu _3}I- \tau {T_1} = 0,\nonumber \\&q{\mu _1}S + q{\mu _2}E + q{\mu _3}I - \tau {T_2} = 0. \end{aligned}$$

let $(E,I,{T_1},{T_2}) = ({E^*},{I^*},{T_1}^*,{T_2}^*)$ satisfy the equality (6), that is,

7 $$\begin{aligned}&\beta S{I^*}{e^{ - \alpha {T_1}^* + \delta {T_2}^*}} - \rho {E^*} = 0,\nonumber \\&\rho {E^*} - \gamma {I^*} = 0,\nonumber \\&p{\mu _1}S + p{\mu _2}{E^*} + p{\mu _3}{I^*} - \tau {T_1}^* = 0,\nonumber \\&q{\mu _1}S + q{\mu _2}{E^*} + q{\mu _3}{I^*} - \tau {T_2}^* = 0. \end{aligned}$$

From the first and the second equation of (7), we obtain

8 $$\begin{aligned} \alpha {T_1}^* - \delta {T_2}^* = \ln \left( \frac{{\beta S}}{\gamma }\right) . \end{aligned}$$

By adding the third and the fourth equation of (7), we get

9 $$\begin{aligned} \alpha \frac{p}{\tau }\left( {\mu _1}S + {\mu _2}{E^*} + {\mu _3}\frac{\rho }{\gamma }{E^*}\right) - \delta \frac{q}{\tau }\left( {\mu _1}S + {\mu _2}{E^*} + {\mu _3}\frac{\rho }{\gamma }{E^*}\right) = \ln \left( \frac{{\beta S}}{\gamma }\right) . \end{aligned}$$

From this we have

10 $$\begin{aligned} {E^*} = \frac{{\ln ({R_0})}}{{\frac{{{\mu _2}}}{\tau }(\alpha p - \delta q) + \frac{{\rho {\mu _3}}}{{\tau \gamma }}(\alpha p - \delta q)}} = \frac{{\tau \gamma \ln ({R_0})}}{{(\alpha p - \delta q)(\gamma {\mu _2} + \rho {\mu _3})}}. \end{aligned}$$

At the same time, we obtain

11 $$\begin{aligned} {I^*} = \frac{\rho }{\gamma }{E^*} = \frac{{\tau \rho \ln ({R_0})}}{{(\alpha p - \delta q)\left( \gamma {\mu _2} + \rho {\mu _3}\right) }}. \end{aligned}$$

By substituting (10) and (11) into the third and the fourth equation of (7), we have

$$\begin{aligned}&p\left[ {\mu _1}S + {\mu _2}\frac{{\tau \gamma \ln ({R_0})}}{{(\alpha p - \delta q)(\gamma {\mu _2} + \rho {\mu _3})}} + {\mu _3}\frac{\rho }{\gamma }\frac{{\tau \gamma \ln ({R_0})}}{{(\alpha p - \delta q)(\gamma {\mu _2} + \rho {\mu _3})}}\right] = \tau {T_1}^*,\\&q\left[ {\mu _1}S + {\mu _2}\frac{{\tau \gamma \ln ({R_0})}}{{(\alpha p - \delta q)(\gamma {\mu _2} + \rho {\mu _3})}} + {\mu _3}\frac{\rho }{\gamma }\frac{{\tau \gamma \ln ({R_0})}}{{(\alpha p - \delta q)(\gamma {\mu _2} + \rho {\mu _3})}}\right] = \tau {T_2}^*. \end{aligned}$$

Simplifying the above equations, we can yield

$$\begin{aligned} {T_1}^* &= \frac{p}{\tau }\left[ {\mu _1}S + {\mu _2}\frac{{\tau \ln ({R_0})(\gamma {\mu _2} + \rho {\mu _3})}}{{(\alpha p - \delta q)(\gamma {\mu _2} + \rho {\mu _3})}}\right] = \frac{p}{\tau }\left[ {\mu _1}S + {\mu _2}\frac{{\tau \ln ({R_0})}}{{(\alpha p - \delta q)}}\right] = \frac{{p\ln ({R_0})}}{{(\alpha p - \delta q)}} + {T_1}^0,\\ {T_2}^* &= {} \frac{q}{\tau }\left[ {\mu _1}S + {\mu _2}\frac{{\tau \ln ({R_0})(\gamma {\mu _2} + \rho {\mu _3})}}{{(\alpha p - \delta q)(\gamma {\mu _2} + \rho {\mu _3})}}\right] = \frac{q}{\tau }\left[ {\mu _1}S + {\mu _2}\frac{{\tau \ln ({R_0})}}{{(\alpha p - \delta q)}}\right] = \frac{{q\ln ({R_0})}}{{(\alpha p - \delta q)}} + {T_1}^0. \end{aligned}$$

So we can obtain endemic equilibrium ${E_*} = ({E^*},{I^*},{T_1}^*,{T_2}^*)$. It is clear that the endemic equilibrium exists if and only if $R_{0}>1$ and $\alpha p - \delta q > 0$. This completes the proof of Theorem 1. $\square$

## Analysis of the model

In this section we will discuss the stability of equilibria of the system (1).

### Stability of the disease-free equilibrium

#### **Theorem 2**

*If*$R_{0} < 1$*and*$\alpha p - \delta q \ge 0$, *then the disease-free equilibrium*$E_{0}$*is globally asymptotically stable*.

#### *Proof*

The characteristic equation of the linearization of system (1) at the disease-free equilibrium $E_{0}$ is

12 $$\begin{aligned} \left| { {\begin{array}{cccc} { - \rho - \lambda }&{}{\beta S{e^{ - \alpha {T_1}^0 + \delta {T_2}^0}}}&{}0&{}0\\ \rho &{}{ - \gamma - \lambda }&{}0&{}0\\ {p{\mu _2}}&{}{p{\mu _3}}&{}{ - \tau - \lambda }&{}0\\ {q{\mu _2}}&{}{q{\mu _3}}&{}0&{}{ - \tau - \lambda } \end{array}} } \right| = 0, \end{aligned}$$

where $\lambda$ is the eigenvalue. Two eigenvalues are $-\tau$ and the other are determined by

$$(\lambda + \rho )(\lambda + \gamma ) - \rho \beta S{e^{ - \alpha {T_1}^0 + \delta {T_2}^0}} = 0.$$

According to (3), the above equation can be rewritten as

13 $${\lambda ^2} + (\rho + \gamma )\lambda + \rho \gamma (1 - {R_0}) = 0.$$

If $R_{0}<1$, then we have

$$\begin{aligned} {\lambda _1} &= {\lambda _2} = - \tau ,\\ {\lambda _3} + {\lambda _4} & = - (\rho + \gamma ) < 0,\\ {\lambda _3}{\lambda _4} &= \rho \gamma (1 - {R_0}) > 0. \end{aligned}$$

It follows from the above equation that all the eigenvalues of (12) are negative. Therefore $E_{0}$ is a locally asymptotically stable equilibrium of (1). We define a Lyapunov function

$$V(t) = E(t) + I(t).$$

It is obvious that $V(t) \ge 0$ and the equality holds if and only if $E(t) = I(t) = 0$. From the third and the fourth equation of (1), we have

$$\begin{aligned} \frac{{d(\alpha {T_1} - \delta {T_2})}}{{dt}} &= \alpha (p{\mu _1}S + p{\mu _2}E + p{\mu _3}I- \tau {T_1}) - \delta (q{\mu _1}S + q{\mu _2}E + q{\mu _3}I - \tau {T_2})\\ &= {\mu _1}S(\alpha p - \delta q) + {\mu _2}E(\alpha p - \delta q) + {\mu _3}I(\alpha p - \delta q) - \tau (\alpha {T_1} - \delta {T_2}). \end{aligned}$$

When $\alpha p - \delta q \ge 0 (\mathrm{i.e.},\alpha {T_1} - \delta {T_2}\ge 0)$, we yield

14 $$\frac{{d(\alpha {T_1} - \delta {T_2})}}{{dt}} \ge {\mu _1}S(\alpha p - \delta q) - \tau (\alpha {T_1} - \delta {T_2}).$$

Using the result of differential inequalities (Lakshmikantham et al. 1988), we obtain

15 $$\begin{aligned} \alpha {T_1} - \delta {T_2} &\ge (\alpha {T_1}^0 - \delta {T_2}^0){e^{\int _0^t {( - \tau )} du}} + \int _0^t {{\mu _1}S(\alpha p - \delta q)} {e^{\int _v^t {( - \tau )} du}}dv \nonumber \\ &= (\alpha {T_1}^0 - \delta {T_2}^0){e^{ - \tau t}} + \frac{{{\mu _1}S}}{\tau }(\alpha p - \delta q)(1 - {e^{ - \tau t}}) \nonumber \\ &= \left[ (\alpha {T_1}^0 - \delta {T_2}^0) - \frac{{{\mu _1}S}}{\tau }(\alpha p - \delta q)\right] {e^{ - \tau t}} + \frac{{{\mu _1}S}}{\tau }(\alpha p - \delta q). \end{aligned}$$

Therefore, $\alpha {T_1} - \delta {T_2} \ge \frac{{{\mu _1}S}}{\tau }(\alpha p - \delta q) = \alpha {T_1}^0 - \delta {T_2}^0$, for all $t \ge 0$. Differentiating *V*(*t*) and using $\beta S{e^{ - \alpha {T_1}^0 + \delta {T_2}^0}} = \gamma {R_0}$, we have

$$\begin{aligned} \frac{{dV(t)}}{{dt}} &= \frac{{dE(t)}}{{dt}} + \frac{{dI(t)}}{{dt}}\\ & = \beta SI{e^{ - \alpha {T_1} + \delta {T_2}}} - \gamma I \\ &= I(\gamma {R_0} - \gamma )\\ &= \gamma I({R_0} - 1) \le 0. \end{aligned}$$

It follows that *V*(*t*) is bounded and non-increasing. Therefore, $\mathop {\lim }\nolimits_{t \rightarrow \infty } V(t)$ exists. Note that $\frac{{dV(t)}}{{dt}} = 0$ if and only if $E(t) = I(t) = 0$, $T_1={T_1}^0$ and $T_2={T_2}^0$. By LaSalle Invariance Principle (LaSalle 1987), the disease-free equilibrium $E_{0}$ is globally attracting when $\alpha p - \delta q \ge 0$ and ${R_0} < 1$. Together with the local asymptotic stability, we show that $E_{0}$ is globally asymptotically stable when $\alpha p - \delta q \ge 0$ and ${R_0} < 1$. This completes the proof of Theorem 2. $\square$

#### *Remark 1*

**Fig. 2:**
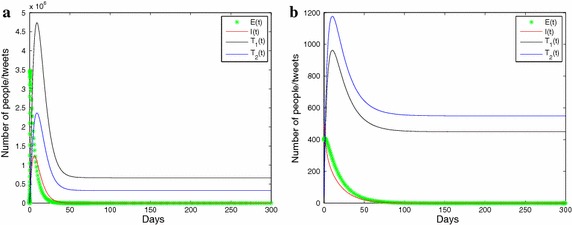
**a** Illustration of disease-free equilibrium of the system (1) is globally asymptotically stable when $R_0<1$ and the positive information more than negative information (i.e., $\alpha p - \delta q>0$, **b** illustration of disease-free equilibrium of the (1) is globally asymptotically stable when $R_0<1$ and the negative information more than positive information (i.e., $\alpha p - \delta q>0$

When $R_{0} < 1$ and $\alpha p - \delta q < 0$, globally asymptotically stability of the disease-free equilibrium $E_{0}$ is not been established. Figure 2b seems to support the idea that the disease-free equilibrium of system (1) is still global asymptotically stable even in this case.

### Stability of the endemic equilibrium

#### **Theorem 3**

*The endemic equilibrium*${E_*}$*is locally asymptotically stable if and only if one of the following statements is satisfied*:

1. $$R_{0}>1$$, $$\alpha p - \delta q > 0$$, and $${\mu _3} \le {\mu _3}^*$$, where $${\mu _3}^* = \frac{{\rho + \tau }}{\rho }{\mu _2}$$;
2. $$R_{0}>1$$, $$\alpha p - \delta q > 0$$, $${\mu _3} > {\mu _3}^*$$, and $$\beta < min\{ {\beta ^*},{\beta ^{**}}\}$$, where $$\beta ^{*}$$*and*$$\beta ^{**}$$*are given by* (25) *and* (27), *respectively*.

#### *Proof*

The characteristic equation of the linearization of system (1) at the endemic equilibrium ${E_*}$ is

$$\begin{aligned} \left| { {\begin{array}{cccc} { - \rho - \xi }&{}{\beta S{e^{ - \alpha {T_1}^* + \delta {T_2}^*}}}&{}{ - \alpha \beta S{I^*}{e^{ - \alpha {T_1}^* + \delta {T_2}^*}}}&{}{\delta \beta S{I^*}{e^{ - \alpha {T_1}^* + \delta {T_2}^*}}}\\ \rho &{}{ - \gamma - \xi }&{}0&{}0\\ {p{\mu _2}}&{}{p{\mu _3}}&{}{ - \tau - \xi }&{}0\\ {q{\mu _2}}&{}{q{\mu _3}}&{}0&{}{ - \tau - \xi } \end{array}} } \right| = 0. \end{aligned}$$

Note that $R_{0}=\frac{{\beta S}}{\gamma }{e^{ - \alpha {T_1}^0 + \delta {T_2}^0}}$ , ${T_1}^* = \frac{{p\ln ({R_0})}}{{(\alpha p - \delta q)}} + {T_1}^0$ and ${T_2}^* = \frac{{q\ln ({R_0})}}{{(\alpha p - \delta q)}} + {T_2}^0$, we have

$$\begin{aligned} \beta S{e^{ - \alpha {T_1}^* + \delta {T_2}^*}} &= \beta S{e^{ - \alpha \left[ \frac{{p\ln ({R_0})}}{{\alpha p - \delta q}} + {T_1}^0\right] + \delta \left[ \frac{{q\ln ({R_0})}}{{\alpha p - \delta q}} + {T_2}^0\right] }} \\ &= \beta S{e^{ - \alpha {T_1}^0 + \delta {T_2}^0}}{e^{\frac{{ - \alpha p + \delta q}}{{\alpha p - \delta q}}\ln ({R_0})}} \\ &= \gamma {R_0}{e^{ - \ln ({R_0})}} \\&= \gamma . \end{aligned}$$

The corresponding characteristic equation becomes

$$\begin{aligned} \left| {{\begin{array}{cccc} { - \rho - \xi }&{}\gamma &{}{ - \alpha \gamma {I^*}}&{}{\delta \gamma {I^*}}\\ \rho &{}{ - \gamma - \xi }&{}0&{}0\\ {p{\mu _2}}&{}{p{\mu _3}}&{}{ - \tau - \xi }&{}0\\ {q{\mu _2}}&{}{q{\mu _3}}&{}0&{}{ - \tau - \xi } \end{array}} } \right| = 0. \end{aligned}$$

Simplifying the above determinant, so the characteristic equation about ${E_*}$ can be rewritten as

16 $$P(\xi ) = {\xi ^4} + {a_1}{\xi ^3} + {a_2}{\xi ^2} + {a_3}\xi + {a_4},$$

where

17 $${a_1} = \rho + \gamma + 2\tau ,$$

18 $$\begin{aligned} {a_2} &= \gamma {I^*}{\mu _2}(\alpha p - \delta q) + {\tau ^2} + 2\tau (\rho + \gamma )\nonumber \\ &= \frac{{\gamma \rho \tau \ln ({R_0}){\mu _2}}}{{\gamma {\mu _2} + \rho {\mu _3}}} + {\tau ^2} + 2\tau (\rho + \gamma ), \end{aligned}$$

19 $$\begin{aligned} {a_3} &= \gamma {I^*}(\alpha p - \delta q)\left[ \rho {\mu _3} + (\tau + \gamma ){\mu _2}\right] + {\tau ^2}(\rho + \gamma )\nonumber \\&= \frac{{\gamma \rho \tau \ln ({R_0})}}{{\gamma {\mu _2} + \rho {\mu _3}}}\left[ \rho {\mu _3} + (\tau + \gamma ){\mu _2}\right] + {\tau ^2}(\rho + \gamma ), \end{aligned}$$

20 $${a_4} = \gamma {I^*}\tau (\alpha p - \delta q)(\gamma {\mu _2} + \rho {\mu _3}) = \gamma \rho {\tau ^2}\ln ({R_0}).$$

It is evident that ${a_i} > 0\,(i = 1,2,3,4)$, when ${R_0} > 1$. On the basis of the Routh–Hurwitz criteria (Murray 1998), we have

$$\begin{aligned} {a_1}{a_2} - {a_3} &= (\rho + \gamma + 2\tau )\left[ \gamma {I^*}{\mu _2}(\alpha p - \delta q) + {\tau ^2} + 2\tau (\rho + \gamma )\right] \\&\quad - \gamma {I^*}(\alpha p - \delta q)\left[ \rho {\mu _3} + (\tau + \gamma ){\mu _2}\right] - {\tau ^2}(\rho + \gamma )\\ &= \gamma {I^*}(\alpha p - \delta q)\left[ (\rho + \tau ){\mu _2} - \rho {\mu _3}\right] + {\tau ^2}(\rho + \gamma + 2\tau ) \\&\quad + 2\tau (\rho + \gamma )(\rho + \gamma + 2\tau ) - {\tau ^2}(\rho + \gamma )\\ &= \gamma {I^*}(\alpha p - \delta q)\left[ (\rho + \tau ){\mu _2} - \rho {\mu _3}\right] + 2\tau {(\rho + \gamma + \tau )^2}\\ &= \frac{{\gamma \rho \tau \ln ({R_0})}}{{\gamma {\mu _2} + \rho {\mu _3}}}\left[ (\rho + \tau ){\mu _2} - \rho {\mu _3}\right] + 2\tau {(\rho + \gamma + \tau )^2}\\ &= \frac{1}{{\gamma {\mu _2} + \rho {\mu _3}}}\{ \gamma \rho \tau \ln ({R_0})\left[ (\rho + \tau ){\mu _2} - \rho {\mu _3}\right] \\&\quad + 2\tau {(\rho + \gamma + \tau )^2}\gamma \rho \tau \ln ({R_0})\}. \end{aligned}$$

Denoting the expression $\gamma \rho \tau \ln ({R_0})[(\rho + \tau ){\mu _2} - \rho {\mu _3}] + 2\tau {(\rho + \gamma + \tau )^2}\gamma \rho \tau \ln ({R_0})$ by $\varphi$, one has

$${a_1}{a_2} - {a_3}=\frac{\varphi }{{\gamma {\mu _2} + \rho {\mu _3}}}.$$

Therefore ${a_1}{a_2} - {a_3}>0$ if and only if $\varphi >0$. Moreover

21 $$\begin{aligned} {a_3}({a_1}{a_2} - {a_3}) - {a_1}^2{a_4} &= \left\{ \gamma {I^*}(\alpha p - \delta q)\left[ \rho {\mu _3} + (\tau + \gamma ){\mu _2}\right] + {\tau ^2}(\rho + \gamma )\right\} \nonumber \\&\quad \times \left\{ \gamma {I^*}(\alpha p - \delta q)\left[ (\rho + \tau ){\mu _2} - \rho {\mu _3}\right] + 2\tau {(\rho + \gamma + \tau )^2}\right\} \nonumber \\&\quad - {(\rho + \gamma + 2\tau )^2}\gamma {I^*}\tau (\alpha p - \delta q)(\gamma {\mu _2} + \rho {\mu _3}) \nonumber \\&= \left[ \rho {\mu _3} + (\tau + \gamma ){\mu _2}\right] \left[ (\rho + \tau ){\mu _2} - \rho {\mu _3}\right] {\left[ \gamma {I^*}(\alpha p - \delta q)\right] ^2} \nonumber \\&\quad + \left\{ \rho \tau {\mu _3}[{(\rho + \gamma )^2} - \tau (\rho + \gamma + 2\tau )] + \gamma \tau {\mu _2}[{(\rho + \gamma )^2} - 2{\tau ^2}] \right. \nonumber \\&\quad \left. + {\tau ^2}{\mu _2}\left[ 2{(\rho + \gamma + \tau )^2} + (\rho + \gamma )(\rho + \tau )\right] \right\} \left[ \gamma {I^*}(\alpha p - \delta q)\right] \nonumber \\&\quad + 2{\tau ^3}(\rho + \gamma ){(\rho + \gamma + \tau )^2}. \end{aligned}$$

Obviously, ${a_3}({a_1}{a_2} - {a_3}) - {a_1}^2{a_4} = 0$ is an unary quadratic equation about $\gamma {I^*}(\alpha p - \delta q)$. With the help of **Matlab**, we can obtain two solutions of this unary quadratic equation, in other words,

22 $$\begin{aligned} {\left( {\gamma {I^*}(\alpha p - \delta q)} \right) _1}&= \frac{{ - (2\rho {\tau ^2} + 2\gamma {\tau ^2} + 2{\tau ^3})}}{{\rho {\mu _3} + (\gamma + \tau ){\mu _3}}}, \end{aligned}$$

23 $$\begin{aligned} {\left( {\gamma {I^*}(\alpha p - \delta q)} \right) _2}&= \frac{{\tau \left[ {{(\rho + \gamma )}^2} + \tau (\rho + \gamma )\right] }}{{\rho {\mu _3} - (\rho + \tau ){\mu _3}}}. \end{aligned}$$

Hence, the endemic equilibrium ${E_*}$ is locally asymptotically stable if and only if $\varphi >0$ and ${a_3}({a_1}{a_2} - {a_3}) - {a_1}^2{a_4}>0$.

Let $${\mu _3}^* = \frac{{\rho + \tau }}{\rho }{\mu _2}$$.

If $${\mu _3} \le {\mu _3}^* ({\rm i.e.,} (\rho + \tau ){\mu _2} - \rho {\mu _3} \ge 0 )$$, then $$\varphi >0$$.

For ${\mu _3} > {\mu _3}^* ({\rm i.e.,} (\rho + \tau ){\mu _2} - \rho {\mu _3} < 0 )$. If $\varphi >0$, then the following inequality needs to be satisfied

24 $$\begin{aligned} ln({R_0}) < \frac{{2\tau {{(\rho + \gamma + \tau )}^2}(\gamma {\mu _2} + \rho {\mu _3})}}{{\gamma \rho \tau [\rho {\mu _3} - (\rho + \tau ){\mu _2}]}}. \end{aligned}$$

Note that $R_{0}=\frac{{\beta S}}{\gamma }{e^{ - \alpha {T_1}^0 + \delta {T_2}^0}}$, we solve $\beta$ and obtain $\beta < {\beta ^*}$, where

25 $$\begin{aligned} {\beta ^*} = \frac{\gamma }{S}{e^{\frac{{2{{(\rho + \gamma + \tau )}^2}(\gamma {\mu _2} + \rho {\mu _3})}}{{\gamma \rho \left[ \rho {\mu _3} - (\rho + \tau ){\mu _2}\right] }}}}{e^{\frac{{{\mu _1}S}}{\tau }(\alpha p - \delta q)}}. \end{aligned}$$

Therefore, we show that ${a_1}{a_2} - {a_3}>0$ if (1) ${\mu _3} \le {\mu _3}^*$ or (2) ${\mu _3} > {\mu _3}^*$ and $\beta < {\beta ^*}$.

Next, we prove that if ${\mu _3} \le {\mu _3}^*$, then ${a_3}({a_1}{a_2} - {a_3}) - {a_1}^2{a_4}>0$ holds constantly. In fact, the right first term of (21) is nonegative constantly, namely,

$$\begin{aligned} \left[ \rho {\mu _3} + (\tau + \gamma ){\mu _2}\right] \left[ (\rho + \tau ){\mu _2} - \rho {\mu _3}\right] {\left[ \gamma {I^*}(\alpha p - \delta q)\right] ^2}\ge 0 \end{aligned}$$

We denote the right second term of (21) to be $\psi [\gamma {I^*}(\alpha p - \delta q)]$, where

$$\begin{aligned} \psi&= \rho \tau {\mu _3}\left[ {(\rho + \gamma )^2} - \tau (\rho + \gamma + 2\tau )\right] + \gamma \tau {\mu _2}\left[ {(\rho + \gamma )^2} - 2{\tau ^2}\right] \\&\quad + {\tau ^2}{\mu _2}\left[ 2{(\rho + \gamma + \tau )^2} + (\rho + \gamma )(\rho + \tau )\right] . \end{aligned}$$

Thanks to ${\mu _3} \le {\mu _3}^*$, then $\psi$ is satisfied the following inequality

$$\begin{aligned} \psi \ge \tau \left\{ \rho {\mu _3}{(\rho + \gamma )^2} + 2\tau {\mu _2}\left[ {(\rho + \gamma )^2} + \tau (\rho + \gamma )\right] + \gamma {\mu _2}{(\rho + \gamma )^2}\right\} >0. \end{aligned}$$

Therefore, the right second term of (21) is negative constantly. Obviously, the right third term $2{\tau ^3}(\rho + \gamma ){(\rho + \gamma + \tau )^2}$ of (21) is also negative constantly. Taking the above fact into consideration, we get ${a_3}({a_1}{a_2} - {a_3}) - {a_1}^2{a_4}>0$ holds constantly if ${\mu _3} \le {\mu _3}^*$. Hence the statements (1) of Theorem 3 are proved.

For ${\mu _3} > {\mu _3}^*$ and $\beta < {\beta ^*}$, if ${a_3}({a_1}{a_2} - {a_3}) - {a_1}^2{a_4}>0$ holds, we must make ${\left( {\gamma {H^*}(\alpha p - \delta q)} \right) _2} < \frac{{\tau [{{(\rho + \gamma )}^2} + \tau (\rho + \gamma )]}}{{\rho {\mu _3} - (\rho + \tau ){\mu _3}}}$ hold constantly.

In this case, the quadratic equation ${a_3}({a_1}{a_2} - {a_3}) - {a_1}^2{a_4} = 0$ about $\gamma {I^*}(\alpha p - \delta q)$ is an opening down, and there are two unequal roots (positive and negative). Therefore, we need ${\gamma {I^*}(\alpha p - \delta q)} < \frac{{\tau [{{(\rho + \gamma )}^2} + \tau (\rho + \gamma )]}}{{\rho {\mu _3} - (\rho + \tau ){\mu _3}}}$ is satisfied. Moreover, we can obtain ${a_3}({a_1}{a_2} - {a_3}) - {a_1}^2{a_4} > 0$. From ${\gamma {I^*}(\alpha p - \delta q)} < \frac{{\tau [{{(\rho + \gamma )}^2} + \tau (\rho + \gamma )]}}{{\rho {\mu _3} - (\rho + \tau ){\mu _3}}}$, we can get $\frac{{\gamma \rho \tau \ln ({R_0})}}{{\gamma {\mu _2} + \rho {\mu _3}}} < \frac{{\tau [{{(\rho + \gamma )}^2} + \tau (\rho + \gamma )]}}{{\rho {\mu _3} - (\rho + \tau ){\mu _2}}}$, that is to say,

$$\begin{aligned} \ln ({R_0}) < \frac{{\left[ {{(\rho + \gamma )}^2} + \tau (\rho + \gamma )\right] (\gamma {\mu _2} + \rho {\mu _3})}}{{\gamma \rho \left[ \rho {\mu _3} - (\rho + \tau ){\mu _2}\right] }}. \end{aligned}$$

It follows from the expression of ${R_0}$ (see (3)) that

$$\begin{aligned} \ln \left( \frac{{\beta S}}{\gamma }\right) < \frac{{\left[ {{(\rho + \gamma )}^2} + \tau (\rho + \gamma )\right] (\gamma {\mu _2} + \rho {\mu _3})}}{{\gamma \rho \left[ \rho {\mu _3} - (\rho + \tau ){\mu _2}\right] }} + \alpha {T_1}^0 - \delta {T_2}^0. \end{aligned}$$

Simplifying the above inequality, we have

26 $$\begin{aligned} \beta < \frac{\gamma }{S}{e^{\frac{{\left[ {{(\rho + \gamma )}^2} + \tau (\rho + \gamma )\right] (\gamma {\mu _2} + \rho {\mu _3})}}{{\gamma \rho \left[ \rho {\mu _3} - (\rho + \tau ){\mu _2}\right] }}}}{e^{\frac{{{\mu _1}S}}{\tau }(\alpha p - \delta q)}}. \end{aligned}$$

Now, we rewritten inequality (26) as $$\beta <\beta ^{**}$$, where

27 $$\begin{aligned} \beta ^{**}=\frac{\gamma }{S}{e^{\frac{{\left[ {{(\rho + \gamma )}^2} + \tau (\rho + \gamma )\right] (\gamma {\mu _2} + \rho {\mu _3})}}{{\gamma \rho \left[ \rho {\mu _3} - (\rho + \tau ){\mu _2}\right] }}}}{e^{\frac{{{\mu _1}S}}{\tau }(\alpha p - \delta q)}}. \end{aligned}$$

Furthermore, we have $${a_3}({a_1}{a_2} - {a_3}) - {a_1}^2{a_4}>0$$ holds, if $${\mu _3} > {\mu _3}^*$$, $$\beta < {\beta ^*}$$ and $$\beta <\beta ^{**}$$, where $$\beta ^{*}$$ and $$\beta ^{**}$$ are given by equality (25) and equality (27) respectively. Owing to $$R_{0}>1$$, combining with (20), we can obtain $$a_4>0$$. Thus we have $${a_3}{a_4}({a_1}{a_2} - {a_3}) - {a_1}^2{a_4^2} > 0$$ if $${a_3}({a_1}{a_2} - {a_3}) - {a_1}^2{a_4} > 0$$. From Routh-Hurwitz criteria (Murray 1998), we know the endemic equilibrium is locally asymptotically stable. This completes the proof of the statements (2) of Theorem 3. $$\square$$

### Hopf bifurcation

#### **Theorem 4**

*A Hopf bifurcation occurrs when*$$\beta$$*increases and the curve*$${\beta ^{**}}$$*is crossed, where*$${\beta ^{**}}$$*is defined in equation* (26).

#### *Proof*

Note that two eigenvalues of the fourth degree characteristic polynomial (16) are always negative. To observe how the real parts of the other two eigenvalues change their signs, we check the transversality condition of the Hopf bifurcation. Assume $P(\xi )$ has two real roots *x*, *y* and a pair of complex roots $a \pm bi$, where $x<0,y<0$ and $a,b \in R$. We yield

$$\begin{aligned} P(\xi )&= (\xi - x)(\xi - y)[\xi - (a + bi)][\xi - (a - bi)]\\&= [{\xi ^2} - (x + y)\xi + xy][{\xi ^2} - 2a\xi + ({a^2} + {b^2})]\\&= {\xi ^4} - (2a + x + y){\xi ^3} + \left[ ({a^2} + {b^2}) + xy + 2a(x + y)\right] {\xi ^2}\\&\quad- \left[ (x + y)({a^2} + {b^2}) + 2axy\right] \xi + xy({a^2} + {b^2}). \end{aligned}$$

Taking equation (16) into consideration, we obtain

$$\begin{aligned} {a_1}&= - (2a + x + y),\\ {a_2}&= ({a^2} + {b^2}) + xy + 2a(x + y),\\ {a_3}&= - \left[ (x + y)({a^2} + {b^2}) + 2axy\right] ,\\ {a_4}&= xy({a^2} + {b^2}), \end{aligned}$$

where ${a_i} > 0\,(i = 1,2,3,4)$ are given in equations (17)–(20). Now we investigate the case when $P(\xi )=0$ has a pair of purely imaginary roots, $\text{i.e.,}\,a=0$. In this case, we get

$$\begin{aligned} {a_1} = - (x + y),\quad {a_2} = {b^2} + xy, \quad {a_3} = - (x + y){b^2}, \quad {a_4} = xy{b^2}. \end{aligned}$$

Thus, $${a_3}({a_1}{a_2} - {a_3}) - {a_1}^2{a_4} = 0$$, which leads to $$\beta = {\beta ^{**}}$$. Therefore, the occurrence of a pair of purely imaginary roots corresponds to the threshold curve $$\beta = {\beta ^{**}}$$. Substituting $$a+bi$$ into the characteristic equation (16), we yield $$P(a + bi) = 0$$. Thus, $$Re[P(a+bi)]=0$$, where *Re* denotes the real part of a complex number. Calculating $$Re[P(a+bi)]=0$$, we have

28 $$\begin{aligned} \Phi&= \mathrm{Re}\left[ {(a + bi)^4} + {a_1}{(a + bi)^3} + {a_2}{(a + bi)^2} + {a_3}(a + bi) + {a_4}\right] \nonumber \\&= {a^4} - 6{a^2}{b^2} + {b^4} + {a_1}({a^3} - 3a{b^2}) + {a_2}({a^2} - {b^2}) + {a_3}a + {a_4} \nonumber \\&= {a^4} - 6{a^2}{b^2} + {b^4} + {a_1}{a^3} - 3{a_1}a{b^2} + {a_2}{a^2} - {a_2}{b^2} + {a_3}a + {a_4} \nonumber \\&= 0. \end{aligned}$$

From (19) and (20), we know that $a_3$ and $a_4$ rely on $\beta$ due to $R_0$ accommodates $\beta$. Consequently, $\Phi (a,\beta )=0$ defines an implicit function $a(\beta )$ with the independent variable $\beta$. Differentiating $\Phi$ in regard to $\beta$, we have $\frac{{\partial \Phi }}{{\partial \beta }} = 0$, which leads to $\frac{{\partial \Phi }}{{\partial a}}\frac{{\partial a}}{{\partial \beta }} + \frac{{\partial \Phi }}{{\partial \beta }} = 0$. Thereby, $\frac{{\partial a}}{{\partial \beta }} = - \frac{{\partial \Phi }}{{\partial \beta }}/\frac{{\partial \Phi }}{{\partial a}}$.

Then, we decide the sign of $$\frac{{\partial a}}{{\partial \beta }}$$ along the curve $$\beta = {\beta ^{**}}$$. Noticing that $$a=0$$ and $${a_2} = {b^2} + xy$$ on the curve $$\beta = {\beta ^{**}}$$, and only $$a_2$$, $$a_3$$ and $$a_4$$ rely upon $$\beta$$. So we have

29 $$\begin{aligned} \frac{{\partial \Phi }}{{\partial \beta }}{|_{\beta = {\beta ^{**}}}}&= \left( {a^2}\frac{{\partial {a_2}}}{{\partial \beta }} - {b^2}\frac{{\partial {a_2}}}{{\partial \beta }} + a\frac{{\partial {a_3}}}{{\partial \beta }} + \frac{{\partial {a_4}}}{{\partial \beta }}\right) {|_{\beta = {\beta ^{**}}}} \nonumber \\&= \left( - {b^2}\frac{{\partial {a_2}}}{{\partial \beta }} + \frac{{\partial {a_4}}}{{\partial \beta }}\right) {|_{\beta = {\beta ^{**}}}} \nonumber \\&= \frac{{\gamma \rho \tau }}{{{\beta ^{**}}}}\left( \tau - \frac{{{b^2}{\mu _2}}}{{\gamma {\mu _2} + \rho {\mu _3}}}\right) . \end{aligned}$$

From (13) we can get

$$\begin{aligned} {\lambda _{3,4}} = \frac{{ - \left( \rho + \gamma \right) \pm \sqrt{4\rho \gamma (1 - {R_0}) - {{\left( \rho + \gamma \right) }^2}} i}}{2}. \end{aligned}$$

Further, we can easily obtain

30 $$\begin{aligned} {b^2} = \rho \gamma (1 - {R_0}) - \frac{{{{\left( \rho + \gamma \right) }^2}}}{4}. \end{aligned}$$

By substituting (30) into (29), we obtain

$$\begin{aligned} \frac{{\partial \Phi }}{{\partial \beta }}{|_{\beta = {\beta ^{**}}}}&= \frac{{\gamma \rho \tau }}{{{\beta ^{**}}}}\left( \tau - \frac{{{b^2}{\mu _2}}}{{\gamma {\mu _2} + \rho {\mu _3}}}\right) \\&= \frac{{\gamma \rho \tau }}{{{\beta ^{**}}(\gamma {\mu _2} + \rho {\mu _3})}}\left[ \tau (\gamma {\mu _2} + \rho {\mu _3}) - {b^2}{\mu _2}\right] \\&= \frac{{\gamma \rho \tau }}{{{\beta ^{**}}(\gamma {\mu _2} + \rho {\mu _3})}}\left[ \tau (\gamma {\mu _2} + \rho {\mu _3}) - \rho \gamma (1 - {R_0}){\mu _2} + \frac{{{{(\rho + \gamma )}^2}}}{4}{\mu _2}\right] > 0, \end{aligned}$$

and

$$\begin{aligned} \frac{{\partial \Phi }}{{\partial a}}{|_{\beta = {\beta ^{**}}}}&= \left( 4{a^3} - 12a{b^2} + 3{a_1}{a^2} - 3{a_1}{b^2} + 2{a_2}a + {a_3}\right) {|_{\beta = {\beta ^{**}}}}\\&= - 3{a_1}{b^2} + {a_3}\\&= 2(x + y){b^2} < 0(x,y < 0). \end{aligned}$$

Thus, we have

$$\begin{aligned} \frac{{\partial a}}{{\partial \beta }}\left| {_{\beta = {\beta ^{**}}}} = - \frac{{\partial \Phi }}{{\partial \beta }}/\frac{{\partial \Phi }}{{\partial a}}\right| {_{\beta = {\beta ^{**}}}} > 0. \end{aligned}$$

This show that a Hopf bifurcation occurs when $$\beta$$ increases and crosses the curve $$\beta = {\beta ^{**}}$$. The proof is completed. $$\square$$

## Numerical simulation

In this section, some numerical results of system (1) are presented for supporting the analytic results obtained above. Our part parameter values on the basis of available data. The incubation time for the 2009 H1N1 influenza pandemic was reported to be between 2 and 10 days with a mean of 6 days (Centers for Disease Control and Prevention (CDC) 2009; Tracht et al. 2011; Pawelek et al. 2014). Thereby, we assume that people in the exposed compartment move to the infectious compartment at a rate $\rho =1/6 day^{-1}$. The infectious period was estimated to be between 4 and 7 days with a mean of 5 days (Leekha et al. 2007; Tracht et al. 2011; Pawelek et al. 2014). Therefore, we choose the recovery rate to be $\gamma =0.2 day^{-1}$. The susceptible population size *S* is set to 1 million and initially 10 people get exposed to the disease (Tracht et al. 2011; Pawelek et al. 2014). The other parameters are chosen to illustrate the theoretical results.

In Fig. 2, we carry out numerical simulations to illustrate the results showed in Theorem 2. Figure 2a illustrates the positive information more than negative information (i.e., $\alpha p - \delta q>0$); the parameter values are $\beta =0.0016 {\rm person}^{-1}\,{\rm day}^{-1}$, $\alpha =0.00011\,{\rm tweet}^{-1}$, $\delta =0.00019\,{\rm tweet}^{-1}$, $\rho =1/6\,{\rm day}^{-1}$, $\gamma = 0.2\,{\rm day}^{-1}$, $p=2/3$, $\mu _1=0.2\,{\rm day}^{-1}$, $\mu _2=0.4\,{\rm day}^{-1}$, $\mu _3=0.8\,{\rm day}^{-1}$, $\tau =0.2\,{\rm day}^{-1}$, $q=1/3$, $R_{0}=0.36$. Figure 2b illustrates the negative information more than positive information (i.e., $\alpha p - \delta q<0$); the parameter values are $\beta =0.00016\,{\rm person}^{-1}\,{\rm day}^{-1}$, $\alpha =0.000068\,{\rm tweet}^{-1}$, $\delta =0.00007\,{\rm tweet}^{-1}$, $\rho =1/6\,{\rm day}^{-1}$, $\gamma =0.3\, {\rm day}^{-1}$, $p=0.45$, $\mu _1=0.2\,{\rm day}^{-1}$, $\mu _2=0.4\,{\rm day}^{-1}$, $\mu _3=0.6\,{\rm day}^{-1}$, $\tau =0.2\,{\rm day}^{-1}$, $q=0.55$, $R_{0}=0.54$. It should be observed, of course, that the disease-free equilibrium is globally asymptotically stable. However, we only theoretically prove the first case (Fig. 2a), which is consistent with our conclusion (Theorem 2); for the second case (Fig. 2b), we graphically elucidated the conclusion.

**Fig. 3:**
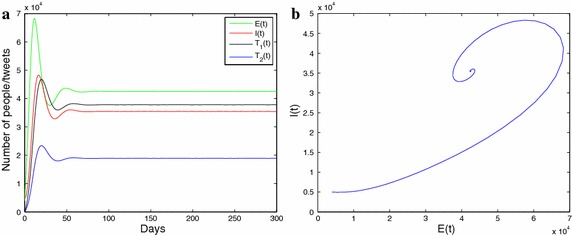
**a** Description of the solution curve under the conditions of Theorem 3.1, and **b** reveals the phase diagram including $E{t}$ and $I{t}$ trajectories under the conditions of Theorem 3.2

**Fig. 4:**
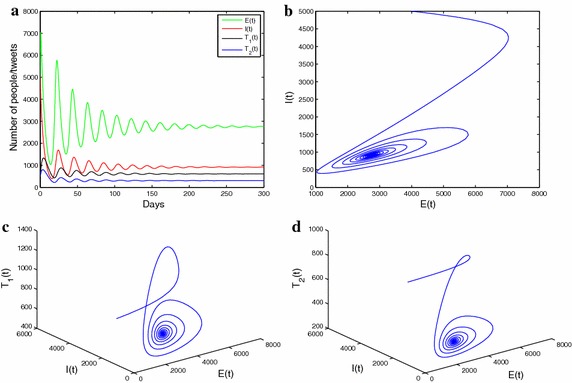
**a** The solution curves of $E_{t}$ , $I_{t}$, $T_1{t}$, $T_2{t}$. **b** the phase diagram including $E_{t}$, and $I_{t}$ trajectories. **c** The phase diagram including $E_{t}$, $I_{t}$ and $T_1{t}$ trajectories. **d** The phase diagram including $E_{t}$, $I_{t}$ and $T_2{t}$ trajectories

**Fig. 5:**
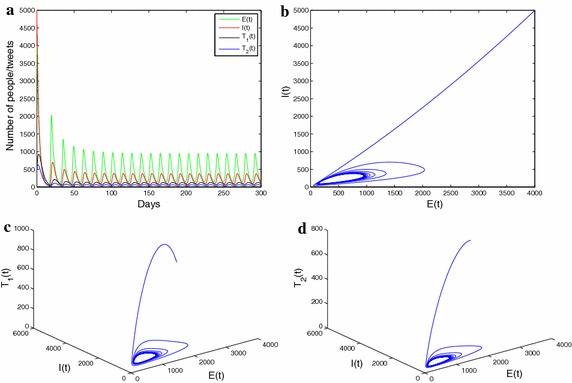
**a** The solution curves of $E_{t}$, $I_{t}$, $T_1{t}$, $T_2{t}$. **b** The phase diagram including $E_{t}$ and $I_{t}$ trajectories. **c** The phase diagram including $E_{t}$, $I_{t}$ and $T_1{t}$ trajectories. **d** The phase diagram including $E_{t}$, $I_{t}$ and $T_2{t}$ trajectories

As is shown in the Figs. 3 and 4, we perform numerical simulations to illustrate the results showed in Theorem 3. Figure 3a describes a graph of the solution curve under the conditions of Theorem 2, and Fig. 3b reveals the phase diagram including *E*(*t*) and *I*(*t*) trajectories under the conditions of Theorem 3. For the purpose of simplicity, we assume $$\mu _1=\mu _2=0$$, and $$\mu _3>0$$; namely, only infectious individuals receive or send positive and negative information about influenza. It can be seen from the Fig. 3a that the results of numerical simulation fit in with the results of the theoretical analysis. Namely, the endemic equilibrium $${E_*}$$ is locally asymptotically stable when $$R_{0}>1$$, $$\alpha p - \delta q > 0$$ and $${\mu _3} \le {\mu _3}^*$$. Figure 4 shows that the endemic equilibrium $$E^{*}$$ is locally asymptotically stable when $$R_{0}>1$$ , $${\mu _3}>{\mu _3}^*$$, and $$\beta <\beta ^{**}$$,

The Fig. 5a–d give information that solution curves of system (1) performs a sustained periodic oscillation and phase trajectories approaches limit cycles. This is not surprising because theoretical derivation achieves the same goal as well. Biologically speaking, the phenomenon sustained periodic oscillation is enforced by unevenly alternating of the positive and negative information during an outbreak of epidemic flu.

## Sensitivity analysis

In this section, we perform sensitivity analysis of reproduction number $R_0$ and compare the effects of several important parameters about Twitter for infectious individuals. To see the effect of $\beta$, $\alpha$, $\delta$, *p*, *q*, and $\tau$ on $R_{0}$, we note that

31 $$\begin{aligned} \frac{{\partial {R_0}}}{{\partial {\mu _1}}}&= - \frac{{\beta {S^2}}}{{\tau \gamma }}(\alpha p - \delta q){e^{ - \frac{{{\mu _1}S}}{\tau }(\alpha p - \delta q)}}, \end{aligned}$$

32 $$\begin{aligned} \frac{{\partial {R_0}}}{{\partial \tau }}&= \frac{{{\mu _1}\beta {S^2}}}{{\gamma {\tau ^2}}}(\alpha p - \delta q){e^{ - \frac{{{\mu _1}S}}{\tau }(\alpha p - \delta q)}}, \end{aligned}$$

33 $$\begin{aligned} \frac{{\partial {R_0}}}{{\partial \beta }}&= \frac{S}{\gamma }{e^{ - \frac{{{\mu _1}S}}{\tau }(\alpha p - \delta q)}}, \end{aligned}$$

34 $$\begin{aligned} \frac{{\partial {R_0}}}{{\partial \gamma }}&= - \frac{{\beta S}}{{{\gamma ^2}}}{e^{ - \frac{{{\mu _1}S}}{\tau }(\alpha p - \delta q)}}, \end{aligned}$$

35 $$\begin{aligned} \frac{{\partial {R_0}}}{{\partial \alpha }}&= - \frac{{p{\mu _1}\beta {S^2}}}{{\gamma \tau }}{e^{ - \frac{{{\mu _1}S}}{\tau }(\alpha p - \delta q)}}, \end{aligned}$$

36 $$\begin{aligned} \frac{{\partial {R_0}}}{{\partial \delta }}&= \frac{{q{\mu _1}\beta {S^2}}}{{\gamma \tau }}{e^{ - \frac{{{\mu _1}S}}{\tau }(\alpha p - \delta q)}}. \end{aligned}$$

**Fig. 6:**
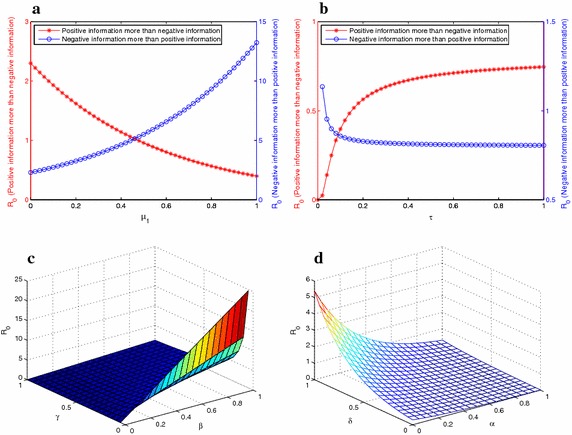
**a** Illustration of the relationship between the basic reproductive $R_{0}$ and $\mu _1$. **b** Illustrates the relationship between the basic reproductive number $R_{0}$ and $\tau$. **c** Illustrates the relationship between the basic reproductive number $R_{0}$, $\beta$ and $\gamma$. **d** Illustrates the relationship between the basic reproductive number $R_{0}$, $\delta$ and $\alpha$

Figure 6a illustrates the relationship between the basic reproductive number $R_0$ and $\mu _1$. According to Eq. (31), if $\frac{{\partial {R_0}}}{{\partial {\mu _1}}}>0$ (i.e. $\alpha p - \delta q<0$), then $R_0$ increases as $\mu _1$ increases, and if $\frac{{\partial {R_0}}}{{\partial {\mu _1}}}<0$ (i.e. $\alpha p - \delta q>0$), then $R_0$ decreases as $\mu _1$ increases. Figure 6b illustrates the relationship between the basic reproductive number $R_0$ and $\tau$. According to Eq. (32), when $\frac{{\partial {R_0}}}{{\partial {\tau}}}>0$ (i.e. $\alpha p - \delta q>0$), $R_0$ increases as $\tau$ increases, and when $\frac{{\partial {R_0}}}{{\partial {\tau}}}>0$ (i.e. $\alpha p - \delta q<0$), $R_0$ decreases as $\tau$ increases. By analyzing Eqs. (33) and (34), Fig. 6c distinctly demonstrates that the greater $\beta$ increases, the more significant $R_0$ grows, and the smaller $\gamma$ decreases, the more remarkable $R_0$ enlarges. Combining Fig. 6d and Eqs. (35) and (36), we can comprehensibly perceive that if $\alpha$ increases, then $R_0$ will decrease, and if $\delta$ increases, then $R_0$ will increase. Biologically, this means that to reduce influence of negative information and transmission rate or increase influence of positive and recover rate are vital essential for controlling influenza.

## Conclusions and discussions

**Fig. 7:**
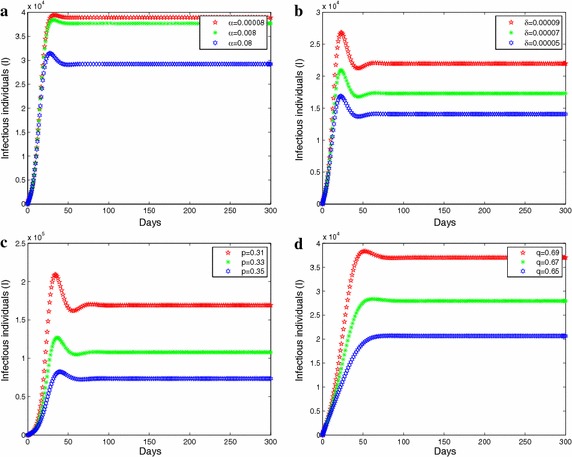
**a** Illustration of the dynamics of infectious individuals with respect to different $\alpha$. **b** Illustration of the dynamics of infectious individuals with respect to different $\delta$. **c** Illustration of the dynamics of infectious individuals with respect to different $p$ . **d** Illustration of the dynamics of infectious individuals with respect to different $q$

First, we will discuss the influence of several important parameters about Twitter to infectious individuals through graphical approach. In Fig. 7a, b, we consider the dynamics of infectious individuals with respect to different factors affecting the spread rate due to positive information (Fig. 7a) and negative information (Fig. 7b). The simulation shows that the upper positive factors lead to the lower infectious cases and the upper negative factors bring about the upper infectious cases. However, under the same conditions, changes in the magnitude of the positive factors is distinctly greater than the negative factors. Thus, as is shown in the Fig. 7a, b, we learn that the impact of negative information on the flu is not so much while it does affect the influenza. In Figs. 7c, d, we research the dynamics of infectious individuals in regard to distinct rates provided positive information (Fig. 7c) and negative information (Fig. 7d). Analyzing it further, we get the same conclusion with the above Figures 7a, b, namely, despite the impact posed the negative information is not significant than the impact caused the positive information on influenza while its impact on the influenza virus is extraordinary.

In the above analysis of the model, we suppose that the number of susceptilbes remains relatively constant. In fact, the number of susceptibles may be obviously decreased due to infection. If we ignore the natural birth and death of susceptibles during an epidemic, the dynamic behavior of *S*(*t*) can be characterized by the following equation

37 $$\begin{aligned} \frac{{dS(t)}}{{dt}} = - \beta SI{e^{ - \alpha {T_1} + \delta {T_2}}}. \end{aligned}$$

The rest of the equations is the same as system (1). The current model has two possible steady states: one is trivial (all the valuables are 0) and the other is the disease-free equilibrium $(S_0, 0,0, \frac{{p{\mu _1}S_0}}{\tau }, \frac{{q{\mu _1}S_0}}{\tau })$, where $S_0$ is the initial value of susceptibles. The analysis of the second steady state is nearly the same as our above analysis for the disease-free equilibrium.

Compared with Pawelek et al. (2014), in this paper we consider the influence on the positive and negative information at the same time. Hence, our model is more closer to real life. In system (1), we only consider the specific case that the number of susceptible people is relatively constant. Generating speaking, this is the idea state. However, if we further consider the recruitment of the susceptible people, we could modify system (1) to the following model:

38 $$\begin{aligned} \frac{{dS(t)}}{{dt}}&= \mu N - \beta SI{e^{ - \alpha {T_1} + \delta {T_2}}},\nonumber \\ \frac{{dE(t)}}{{dt}}&= \beta SI{e^{ - \alpha {T_1} + \delta {T_2}}} - \rho L,\nonumber \\ \frac{{dI(t)}}{{dt}}&= \rho E - \gamma I,\nonumber \\ \frac{{d{T_1}(t)}}{{dt}}&= p{\mu _1}S + p{\mu _2}E + p{\mu _3}I - \tau {T_1},\nonumber \\ \frac{{d{T_2}(t)}}{{dt}}&= q{\mu _1}S + q{\mu _2}E + q{\mu _3}I - \tau {T_2}, \end{aligned}$$

where $\mu$ represents the recruitment rate of the susceptible people. We leave these works for the future.
